# Electron pressure drives THz phonons in metal–metal superlattices

**DOI:** 10.1038/s41467-026-73927-y

**Published:** 2026-06-16

**Authors:** Jan-Etienne Pudell, Maximilian Mattern, Marc Herzog, Alexander von Reppert, Chandan K. Singh, Daniel Schick, Michel Hehn, Ulrike Boesenberg, Angel Rodriguez-Fernandez, Roman Shayduk, Wonhyuk Jo, Johannes Möller, Jörg Hallmann, James Wrigley, Peter M. Oppeneer, Anders Madsen, Matias Bargheer

**Affiliations:** 1 European X-ray Free-Electron Laser Facility, Schenefeld, Germany; 2 Institut für Physik und Astronomie, Universität Potsdam, Potsdam, Germany; 3 Max-Born-Institut (MBI) im Forschungsverbund Berlin e.V., Berlin, Germany; 4 Department of Physics and Astronomy, Uppsala University, P.O. Box 516, Uppsala, Sweden; 5 Institut Jean Lamour (UMR CNRS 7198), Université Lorraine, Nancy, France; 6 Helmholtz-Zentrum Berlin für Materialien und Energie GmbH, Wilhelm-Conrad-Röntgen Campus, BESSY II, Berlin, Germany

**Keywords:** Metamaterials, Acoustics, Electronic properties and materials

## Abstract

Ultrafast control of lattice motion in metals is a central challenge for high-frequency strain engineering and spintronic applications. Coherent strain control at terahertz (THz) frequencies in metals has remained elusive because free electrons are expected to delocalize energy beyond the optical penetration depth, preventing rapid and efficient stress generation. Here we show that robust and cost-effective metal–metal superlattices (SLs), where periodic repetitions of bilayers — each layer a few atoms thick — are deposited by sputtering, constitute thermoacoustic metamaterials that overcome this limitation. We combine femtosecond X-ray diffraction with mode-resolved density-functional theory and two-temperature modeling to show that electron pressure, rather than phonon stress, drives a large-amplitude coherent terahertz (1 THz) lattice oscillation in sputtered Pt/Cu superlattices. We establish electron pressure as an engineerable, dominant actuation mechanism in metallic metamaterials which can be tailored by the pitch and the constituent materials of the sputtered SL structure, enabling applications such as ultrafast strain-mediated antiferromagnetic spintronic devices.

## Abstract (web summary)

All-metallic superlattices act as acoustic metamaterials, where electron pressure drives large-amplitude  ~ 1 THz coherent phonons after femtosecond photo excitation, tunable via layer periodicity for ultrafast strain and spintronic applications.

## Introduction

Optical control of nanoscale metallic heterostructures is relevant to technological applications, including spin dynamics^1–3^, photocatalytic/photothermal^4^, and plasmonic chemistry^5–7^. Strain engineering and THz excitations are promising frontiers, however driving specific tailored terahertz (THz) frequency components of strain in metals remains challenging because free electrons are expected to delocalize optical energy beyond the penetration depth, preventing rapid localized stress formation with high wavevector components. Technologically, metal–metal superlattices, composed of nanometric bilayers of dissimilar metals, offer a class of thermoacoustic metamaterials in which electronic and elastic properties can be tailored at the atomic scale. In magnetic device design, metal superlattices (SLs) are ubiquitous^8–14^. Electron transport across metal–metal interfaces plays a crucial role in metal heterostructures, underpinning both electro-optical^15^ and magnetic devices such as giant magnetoresistance stacks^16^, spin valves^17^, and THz spintronic emitters^1^.

All-metallic superlattices are straightforward to fabricate by sputtering. They would provide a robust platform for converting optical excitation into specific coherent THz lattice motion, provided that steep gradients of stresses could be rapidly formed. A single metal layer would produce a broadband wavepacket to form the sharp strain gradient of a bipolar strain pulse emitted by the sample surface^18,19^. Guided by diffusive two-temperature models (d2TMs) that distinguish electrons and phonons as carriers of heat^20^ to describe ultrafast energy transport^21–23^, heterostructures have enabled control of precession^24^ or switching^2^ of magnetization. On the fundamental physics side, intriguing inverted temperature gradients^4^, heat transport without heating^25^ and dominant conduction of heat into noble metals by phonons^26^ were revealed. The electron-phonon coupling constant *g* dictates the spatial distribution and temporal scale of energy transfer to the phonon system^27,28^, and density-functional theory (DFT) predicts local, layer-dependent *g* values for the incoherent transfer of electronic energies to phonons.

Experiments using optical probes average over the optical penetration depth and cannot resolve buried metallic layers. Therefore, only very few experiments have investigated the ultrafast strain in metal–metal SLs on ultrafast timescales^29–33^. This can nowadays be achieved by ultrafast X-ray diffraction (UXRD) experiments, which, in contrast, provide material-specific and depth-resolved information on energy transport even in opaque materials^34,35^. Electron pressure can be the dominant stress on sub-picosecond timescales. Already in semiconductors, photoexcited carriers can propagate and thereby generate stress profiles that deviate from the optical absorption profile^34,36^. In metals, however, rapid electronic transport delocalizes the deposited energy and suppresses steep electronic-stress gradients. For non-metallic multilayer systems^37^ and at metal surfaces^38–42^, the pressure gradients at the interfaces efficiently drive structural motion. Whether electronic stress gradients can form across purely metallic interfaces remains unclear because experimental evidence is missing. In metal heterostructures, electrons are generally considered free, yet potential steps and band-structure mismatch between different metals could localize electronic states. If this localization were strong enough, it could generate an electron-pressure gradient at the interface between two metals capable of driving coherent lattice oscillations in a fully metallic superlattice.

Here we demonstrate that electronic stress in a metal–metal superlattice triggered by optical excitation efficiently generates large amplitude strain waves at 1 THz, where the frequency is engineered by periodic sputtering of 2.5 nm Pt and 2.3 nm Cu, opening future perspectives for this meta-material. We use ultrafast X-ray diffraction (UXRD) to resolve a 1 THz strain wave with a giant 1% strain amplitude, confirming a narrow-band phonon mode with a wavelength below the optical skin depth. To actuate a coherent and narrowband strain wave with *T* = 1 ps period efficiently and with large amplitude, the spatially periodic stress modulation inside the SL must develop much faster than *T*, which is faster than the effective time scale *τ*_eff_ ≈ 1 ps of incoherent energy relaxation between electrons and phonons in these metals. The electronic stress gradient is tailored by the spatial period of the metamaterial, which has a spatially varying density of states *D*(*E*_F_) at the Fermi level dictated by the local band structure. The observed zero-phase, large-amplitude strain wave can only originate from the instantaneous electron pressure localized in the Pt layers before energy transfer to the phonons occurs. To substantiate this conclusion, we provide a model of the observed time-dependent X-ray diffraction signal that identifies electron pressure as the main driver. Ab-initio calculations of mode-resolved electron-phonon coupling for Pt and Cu, derived from density-functional theory (DFT), confirm that the electron-phonon coupling is too slow to contribute significantly to coherent THz phonon generation via phonon stress.

These results suggest that such high-frequency modulation of longitudinal strain in robust sputtered metallic heterostructures could enable future devices in which quasiparticles in the THz range are addressed via strain to effectuate, e.g., superconductivity or spin dynamics. With the SL period approaching a few atomic layers, opto-acoustic design can be added to metamaterials such as synthetic ferrimagnets^43^.

## Results

**Fig. 1:**
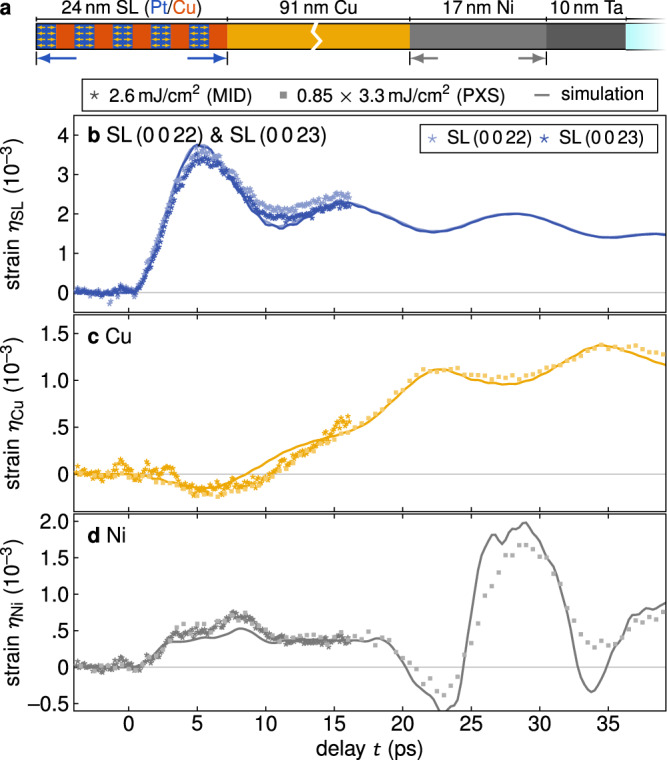
Transient lattice strain evidenced by Bragg peak shifts. **a** Schematic of the Pt/Cu superlattice (SL) on a thick Cu and a thin Ni layer. The blue/gray arrows at the bottom indicate the average expansion of the SL and of the Ni layer. The small orange arrows inside Pt indicate additional motion within the SL. **b** Transient average strain *η*_SL_ of the SL and **c**, **d** transient strain *η*_Cu_ of the Cu and *η*_Ni_ of the Ni layer, respectively, for an absorbed excitation fluence of 2.6 mJ/cm^2^. **c**, **d** contain data from the Materials Imaging and Dynamics (MID) instrument and the plasma X-ray source (PXS) setup (see “Methods”). The data of the PXS measurements are scaled by a factor of 0.85 to match the different absorbed fluence of the MID measurement. They provide an additional validation that the model agrees with the data at longer delays. The lines originate from a simulation considering diffusion according to a diffusive two-temperature model (d2TM) coupled to elastic wave propagation (see text).

To exemplify electron-pressure driven large amplitude actuation of a narrow-band 1 THz wave, we designed the metallic multilayer sample depicted in Fig. 1a (for details see the “Methods”). The sample is excited with a femtosecond laser pulse at a wavelength of 800 nm. The optical energy is absorbed inhomogeneously along the depth, mainly in the Pt layers of the SL. Figure 1 shows the resulting transient average out-of-plane lattice strains, i.e., the relative change of the lattice constant. They are determined from the transient change of the Bragg peak positions along the out-of-plane coordinate *q*_*z*_ in reciprocal space. The metamaterial’s strain *η*_SL_ (Fig. 1b) can be equivalently identified by its (0 0 22) and (0 0 23) SL Bragg reflections, which are close to the (1 1 1) reflections of the constituting elements (see “Methods”: the metamaterial’s unit cell has a large out-of-plane lattice constant leading to high Miller indices in the X-ray diffraction). The strains *η*_Cu_ and *η*_Ni_ for the thicker Cu and Ni layers are shown in Fig. 1c, d, respectively. As expected, both SL peaks show identical transient relative shifts, which are a measure of the average artificial lattice constant. The maximum shift occurs at 5 ps, when the expansive strain wave starting at the surface has propagated through the entire metamaterial, at the corresponding sound velocities *v*_s,Pt_ and *v*_s,Cu_, neglecting the small phononic bandgap at the edge of the mini-Brillouin zone^32,44^. The strain responses of the buried Cu and Ni layers (Fig. 1c, d) display the ultrafast diffusion of hot electrons through the thick Cu layer towards the Ni layer, where the energy is very efficiently coupled to phonons^25^. The weak electron-phonon coupling and concomitant large electronic heat conductivity in Cu enable rapid electronic energy transport from the photoexcited SL to the Ni layer, keeping the Cu phonons cold, which even enhances the electronic heat transport. The nearly simultaneous expansion of the Ni film and the SL causes compression of the Cu layer in the first few picoseconds. Subsequently, the Cu layer expands within tens of picoseconds according to its thermoelastic response^25^. In particular, the early Cu and Ni dynamics show that the electrons are indeed free to rapidly move through both the metamaterial and the Cu layer and further into the Ni, where they are dissipated to phonons. Nonetheless, they also effectively actuate the coherent 1 THz mode as we show in the following.

The combination of layer thicknesses *d* and sound velocities *v*_s_ of the Pt/Cu SL considered here results in a frequency of the fundamental SL phonon mode^37,44^ of nearly 1 THz:

1 $${\nu }_{{{{\rm{SL}}}}}={\left(\frac{{d}_{{{{\rm{Cu}}}}}}{{v}_{{{{\rm{s}}}},{{{\rm{Cu}}}}}}+\frac{{d}_{{{{\rm{Pt}}}}}}{{v}_{{{{\rm{s}}}},{{{\rm{Pt}}}}}}\right)}^{-1}\approx 0.96\,{{{\rm{THz}}}}.$$

**Fig. 2:**
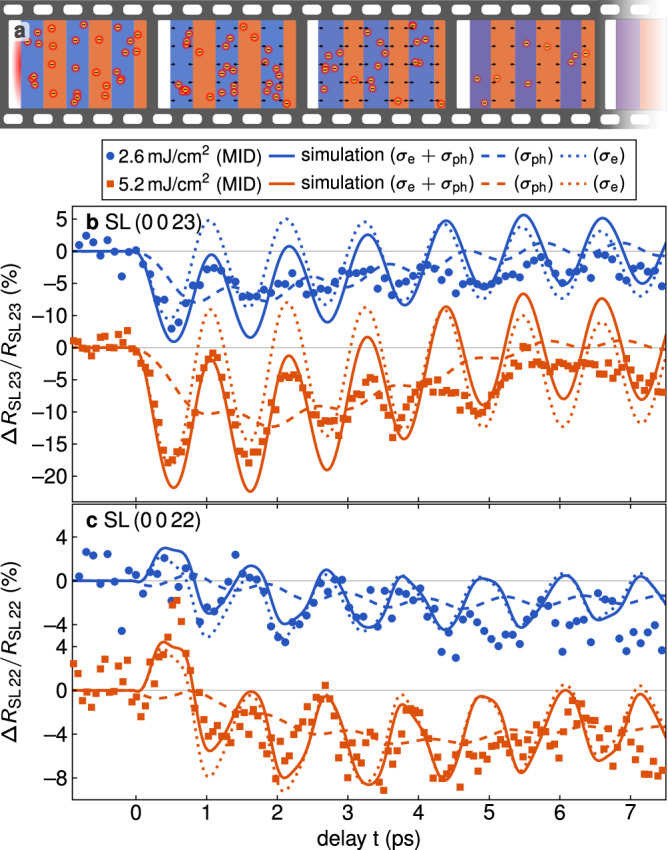
THz phonon driven by electron pressure. **a** Schematic of the photoexcited superlattice (SL) metamaterial: The optical laser pulse excites the electrons within the metal stack. The energy (symbolized by red circles around —) of the electrons rapidly localizes in the Pt layers, exerting pressure on the interfaces that drives the expansion of Pt and, consequently, the compression of Cu. The elastic response leads to oscillations. **b** Transient intensity change Δ*R*_SL23_/*R*_SL23_ of the (0 0 23) SL Bragg peak measured at the Materials Imaging and Dynamics (MID) instrument for two different absorbed fluences (2.6 mJ/cm^2^ [blue] and 5.2 mJ/cm^2^ [orange]). **c** same for (0 0 22). The lines originate from a simulation of the elastic response to the stresses *σ*_e_ (dotted) from electrons and/or *σ*_ph_ (dashed) from phonons, and finally to the sum of both stresses (solid lines). Taking into account both contributions yields the best agreement. The large amplitude of the 1 THz oscillation is only reproduced if a significant electron stress is included, whereas phonon stress mainly dominates the slow, non-oscillatory response. The simulation assumes a perfect sample. Including a phenomenological phonon damping or dephasing would yield an even better match to the measured data.

To resolve the sub-ps changes of the Bragg peak intensity and position, we applied ultrashort hard X-ray pulses from the Materials Imaging and Dynamics (MID) instrument at the European X-ray Free-Electron Laser Facility (EuXFEL)^45^. The oscillatory changes of both SL Bragg peak intensities in Fig. 2 are a direct and quantitative measure of the structural dynamics of the metamaterial, as they correspond to the structure factors of the artificial unit cell^37,46^. Figure 2b, c compares the relative intensity changes of the (0 0 22) and (0 0 23) SL Bragg peaks for two absorbed fluences. The (0 0 23) peak shows a large intensity reduction of 15% within about 0.5 ps, which—according to the metamaterial’s structure factor—reports the maximal 1% expansion of the Pt layers within the SL that simultaneously compresses the Cu layers. The first half-period of the 1 THz oscillation starts immediately after excitation, i.e., with nearly zero phase shift, indicating that the driving stress is quasi-instantaneous. The (0 0 22) Bragg peak essentially confirms this analysis. It oscillates at the same frequency and phase, however, with the opposite sign—even increasing the intensity initially, which is reproduced by the modeling (see “Methods”).

**Fig. 3:**
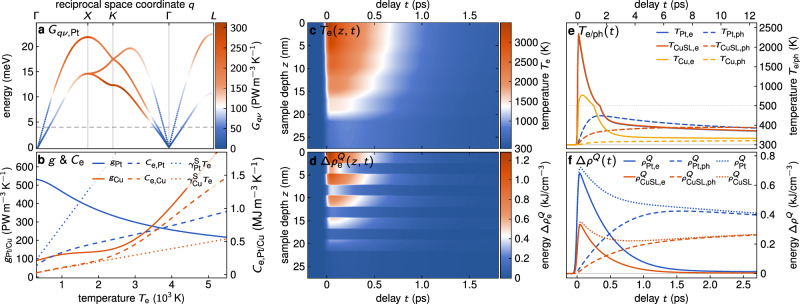
Calculated electron-phonon coupling and results of the diffusive two-temperature model (d2TM). **a** Phonon dispersion of Pt with color-coded mode-specific electron-phonon coupling parameter *G*_*qν*_. The dashed horizontal line indicates the energy of the fundamental superlattice (SL) phonon mode at 1 THz. **b** Mode integrated electron-phonon coupling *g* (solid lines) and electronic heat capacity *C*_e_ (dashed) for Pt and Cu as a function of the electron temperature according to DFT results. The dotted lines represent a Sommerfeld model *C*_e_ = *γ*^S^*T*_e_ for comparison. **c** Electron temperature within the SL for an excitation of 2.6 mJ/cm^2^ (absorbed) with a pulse duration of 50 fs. **d** Corresponding energy density change of electrons $\Delta {\rho }_{{{{\rm{e}}}}}^{Q}$. (Electron and phonon temperatures and energy densities for the full heterostructure are shown in Supplementary Fig. 1). **e** Averaged temperature of the Pt and Cu layer of the SL and the thick Cu layer: The electrons of the SL cool to the Pt and also to the Cu phonon system within the first picoseconds. For a short time, the electrons of the SL are colder than the phonons, because the electron system also loses energy by diffusive transport to the thick Cu layer. For the entire heterostructure, it takes more than 100 ps to equilibrate. Note the change in the scaling of the *y*-axis by a factor of 5 at 500 K. **f** The energy densities of Pt and Cu in the SL after excitation: The difference in the electron system is responsible for the excitation of the engineered THz phonon mode.

The mere observation of the coherent THz modulation starting with zero time delay indicates that it is electronic pressure *σ*_e_ that drives the coherent lattice motion in the metallic heterostructure. It is not the phonon stress *σ*_ph_, which rises on the timescale *τ*_eff_ ≈ *C*_e_(*T*_e_)/*g*(*T*_e_) according to the energy transfer to phonons in the framework of the d2TM^28,34,35^. The specific heat *C*_e_(*T*_e_) increases with temperature and thus fluence, yielding long timescales for the high excitation fluences used here. To provide a microscopic perspective, we performed a first-principles analysis of the mode-specific electron-phonon coupling *G*_*qν*_ (see Fig. 3a). The calculated *G*_*qν*_ reveals that in Pt the energy loss of the electron system is dominated by high-frequency acoustic modes near the Brillouin-zone boundary (~4 THz), whereas the long-wavelength modes around 1 THz contribute less. The mode-specific couplings *G*_*qν*_(*T*_e_) and the mode-averaged coupling *g*(*T*_e_) depend on the electron temperature, yielding an effective incoherent energy-transfer time of *τ*_eff_ > 1 ps for Pt and Cu, consistently with existing literature values^47^. Figure 3a clearly shows that the intrinsic 1 THz modes of Pt and Cu are even more weakly coupled. Therefore, the coherent 1 THz strain wave observed here cannot originate from conventional incoherent electron-phonon energy transfer, but must be driven by sub-picosecond electronic stress (electron pressure) that develops before substantial phonon heating.

Additional experimental cross-checks confirm this analysis: A larger electron-phonon coupling in the Pt layers that could rationalize the excitation of the THz modulation of the SL would reduce the amount of hot electrons reaching the Ni detection layer and increase the mean strain of the SL measured by the peak shift, which would be in disagreement with the measured data in Fig. 1. The electron-phonon coupling time of 1 ps is also in agreement with optical pump-probe measurements performed with this sample.

### Modeling of ultrafast X-ray diffraction (UXRD) data

To identify the driving mechanism of the observed THz oscillations, we modeled the UXRD response using a spatially-resolved diffusive two-temperature framework coupled to elastic wave propagation (see “Methods”).

Although temperatures control the diffusive flow of energy, the relevant quantity for driving coherent longitudinal acoustic strain is the spatiotemporal modulation of the electronic and phononic stresses $\sigma (z,t)={\sigma }_{{{{\rm{e}}}}}(z,t)+{\sigma }_{{{{\rm{ph}}}}}(z,t)={\Gamma }_{{{{\rm{e}}}}}\Delta {\rho }_{{{{\rm{e}}}}}^{Q}(z,t)+{\Gamma }_{{{{\rm{ph}}}}}\Delta {\rho }_{{{{\rm{ph}}}}}^{Q}(z,t)$, which are proportional to the respective energy density changes $\Delta {\rho }_{{{{\rm{e}}}}}^{Q}$ and $\Delta {\rho }_{{{{\rm{ph}}}}}^{Q}$ in the subsystems^34,36^. The proportionality is given by the Grüneisen parameters Γ_e/ph_, which we consider constant for simplicity. Figure 3e, f separates the temperatures and energy density changes in Pt and Cu into electron and phonon contributions. The electron contribution $\Delta {\rho }_{{{{\rm{e}}}}}^{Q}$ in Pt dominates the SL energy density by far within the first picosecond, where the coherent THz phonon is driven. Figure 3c shows a spatiotemporal temperature map reflecting the very fast electron temperature equilibration (Fig. 3e) of Pt and Cu components of the SL within less than 100 fs. The sharp spatial pattern of transient electronic energy density change in Fig. 3d is solely a consequence of the large *D*(*E*_F_) in Pt. Remarkably, the phonon temperatures within the SL exceed all electron temperatures after 5 ps, as electronic heat is dissipated efficiently via the Cu layer. For the entire metal heterostructure, it takes more than 100 ps to equilibrate^35^. Our model predicts that almost all the deposited energy is initially in the Pt electron system, where it produces the stress driving the 1 THz oscillation. After 1 ps, 40% of the energy is transferred to the Pt phonon system, while 10% remain in the Pt electron system. However, a large fraction of the energy (30%) has left the SL and moved towards the thick Cu or Ni layer. This reduces the phonon driven expansion of the SL.

Although our model uses exclusively well-established concepts and thermophysical parameters known from the literature or calculated ab initio by DFT, we explore a rarely investigated realm of physics: The energy confinement in an ultrathin metal heterostructure that constitutes a metallic metamaterial, in which THz phonons are driven by light absorption. In order to show the precision with which the modeling reproduces our experimental data, we have added the model calculations to all the experimental graphs of Figs. 1, 2 as solid lines, using a single set of modeling parameters for all experiments.

Since the relation between stress and strain and the elastic wave equation is linear in this model, we can separately investigate the response to electron stress (dotted lines) and to phonon stress (dashed lines) in Fig. 2b, c. The superposition of both stresses reproduces the data very well for both SL peaks and both fluences with the same set of parameters. The phonon stress alone would drive the THz phonon with a very small amplitude and with a phase shift of several hundred femtoseconds due to the slow average electron-phonon coupling. On the other hand, the electron stress alone does not reproduce the slower components of the intensity modulation, because energy is lost to the phonon system. The fast modulation of the signal at 1 THz thus essentially originates from the electron pressure on the metal–metal interfaces.

## Discussion

Finally, we discuss how the electron pressure can act on the THz strain wave—often referred to as coherent phonon—on a timescale faster than the electron-phonon coupling, which measures how fast energy is transferred incoherently from electrons to phonons. The electron pressure gradient transfers primarily momentum rather than internal energy to coherent lattice motion. In the free-electron picture, the electron stress *σ*_e_ originates from the electronic free energy *F*_e_(*V*, *T*_e_) as ${\sigma }_{{{{\rm{e}}}}}=-{\frac{\partial {F}_{{{{\rm{e}}}}}}{\partial \eta }| }_{{T}_{{{{\rm{e}}}}}}.$ A spatial gradient of this stress produces a force density *f*_e_ = − ∂*σ*_e_/∂*z* that accelerates the lattice in the out-of plane direction, launching a coherent acoustic wave. This process represents a direct momentum exchange between the electronic subsystem and the ions through the stress field, while the electronic energy remains largely within the electron gas. It diffuses to the lower-lying layers and is dissipated by electron-phonon coupling within time *τ*_eff_. In contrast, phonon pressure arises from the anharmonic lattice free energy *F*_ph_(*V*, *T*_ph_) and builds up slowly on the timescale of *τ*_eff_.

In conclusion, we have directly observed the optical excitation of a coherent THz strain wave in a sputtered metal–metal superlattice metamaterial, and quantitatively determined its giant 1% lattice modulation. Ultrafast X-ray diffraction provides direct evidence that this coherent oscillation is driven by electron pressure within the metallic layers, as the intrinsic electron-phonon coupling in Pt and Cu is far too slow to account for the observed amplitude and phase. This establishes a quantitative benchmark for disentangling electron- and phonon-driven energy transfer and stress generation in nanoscale metals. Unlike in earlier experiments, the Sommerfeld constant, rather than the electron-phonon coupling strength^4^, governs the localization of energy and concomitant stress formation. The crucial conclusion is that, irrespective of excitation wavelength and penetration depth of the optical light, rapid ballistic and diffusive transport of electronic energy and electron-electron scattering localizes this electronic energy in the Pt layers of the metamaterial, because the large electron density of states at the Fermi level *D*(*E*_F_) yields a large and localized specific heat contribution. The central design element for such metallic THz phonon metamaterials is the combination of metals with substantially different *D*(*E*_F_), leaving plenty of design parameters such as SL period, asymmetry of the layer thickness, interband excitations or coupling to plasmonic resonances. Our findings call for refined theoretical treatments of non-equilibrium electronic stress in metallic heterostructures, linking models of ultrafast superdiffusion, charge transport in nanoscale metals^21,22,48^ and lattice expansion^49^.

More broadly, our results demonstrate that tailoring electronic and phononic energy transport in artificial nanostructures fabricated by simple sputtering enables the creation of metamaterials with emergent dynamical functionalities. This concept opens routes to magnetoacoustic devices capable of driving antiferromagnetic THz magnons^50,51^, and to strain-based modulation of plasmons^52^, excitons^53,54^, spintronic currents^55^, and correlated electronic phases^56^ beyond the GHz regime.

## Methods

### Sample growth and characterization

**Fig. 4:**
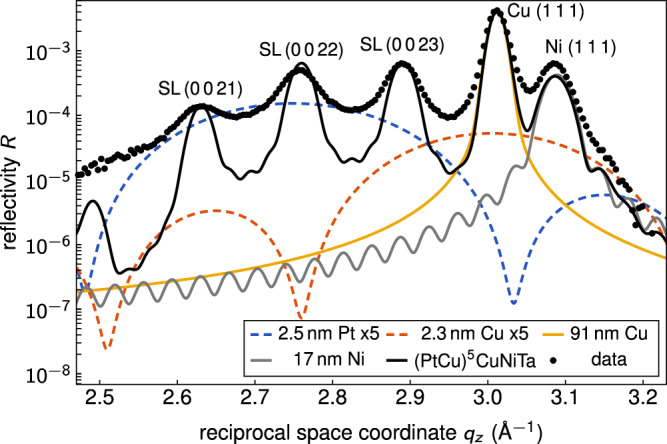
Sample characterization and simulated X-ray reflectivity. Measured XRD reflectivity (black dots) with the Cu, Ni and SL Bragg peaks identified by the modeling (solid black line). The thin lines are calculated diffraction intensities for individual Cu (orange) and Ni (gray) layers. The positions of the SL Bragg peaks are determined by the thicknesses of the SL layers. Their dynamics, i.e., intensity change in a UXRD experiment, are given by the interference of X-rays reflected from the 2.5 nm Pt and 2.3 nm Cu single-layers, which is encoded by shifts of the single-layer diffraction curves (blue and orange dashed). Their intensity is scaled by the number of double layers, i.e., 5.

The metallic multilayer sample consists of an SL with five double layers of 2.5 nm Pt and 2.3 nm Cu on top of a 91 nm Cu layer and a 17 nm Ni layer sputtered on a glass substrate (Corning 1737 AMLCD) with a 10 nm amorphous Ta adhesion layer. A schematic of the sample is shown in Fig. 1a. The short-period SL is needed to investigate energy transport within the SL and generate the THz strain waves. The thick Cu and Ni layers serve to rapidly extract heat and as a cross-check for modeling the heat transport in the framework of a diffusive two-temperature model (d2TM)^25^. The static X-ray diffraction reveals a (1 1 1)-orientation of the Pt, Cu and Ni layers. The calculated diffraction curves of a single Pt (blue) or Cu (orange) layer shown in Fig. 4 determine the intensity of the individual SL Bragg peaks^57^. The good fit of the model to the XRD data confirms the layered crystalline textured, polycrystalline in-plane structure of the sample and yields the individual layer thicknesses and lattice parameters with a few percent accuracy. SL phonon modes near the *Γ*-point emerge from the backfolding of the acoustic phonon dispersion into a mini-Brillouin zone given by the SL period of a SL (here 4.8 nm)^44^.

### Static X-ray diffraction

The Bragg peaks of the SL and the thin films are detected by symmetrically and asymmetrically diffracted X-rays on an area detector (Dectris PILATUS 100K), while the sample and detector are scanned in a classical specular *θ* – 2*θ* scheme. The X-rays were generated by a Cu K_*α*_ X-ray tube and focused with a Montel optic. The measured intensity as a function of the out-of-plane reciprocal space coordinate *q*_*z*_ in Fig. 4 displays two material-specific (1 1 1) Bragg peaks, which are located at *q*_*z*,Cu_ = 3.01 Å^−1^ and *q*_*z*,Ni_ = 3.09 Å^−1^, and three Bragg peaks related to the SL at *q*_*z*,SL 21_ = 2.63 Å^−1^, *q*_*z*,SL 22_ = 2.76 Å^−1^ and *q*_*z*,SL 23_ = 2.89 Å^−1^. They originate from the artificial spatial bilayer periodicity with the artificial SL lattice constant *c*_SL_ = *d*_Pt_ + *d*_Cu_ = 4.8 nm of the metamaterial. The orders 21, 22, and 23, respectively, of the (0 0 1)-oriented SL were extracted from their *q*_*z*_ position and confirmed by a dynamical X-ray diffraction simulation^37,46^. The adjacent SL peaks, SL (0 0 24) and SL (0 0 25) are covered by strong (1 1 1) Bragg reflections from Cu and Ni, respectively.

### Dynamical structure factor change

The intensities of the SL Bragg peaks change as the Pt layers expand and squeeze the adjacent Cu. They are determined by the structure factor of the artificial unit cell (2.5 nm Pt + 2.3 nm Cu) of the metamaterial, which changes as the atoms move within this artificial unit cell. The intensity change can be described by the shift of the calculated single-layer diffraction curves shown in Fig. 4: The expansion of Pt shifts the corresponding envelope function, which is the broad diffraction pattern of a single Pt layer (blue line in Fig. 4), to smaller *q*_*z*_. The concomitant compression of Cu shifts the Cu envelope (orange) to larger *q*_*z*_. Both envelope shifts are responsible for a reduction of the peak intensity of SL (0 0 23), but only the Cu envelope shift significantly increases the intensity of the SL (0 0 22) peak. This peak occurs at the maximum of the Pt envelope, where its shifts yield a minimal intensity change. Finally, the SL Bragg peaks themselves shift to smaller *q*_*z*_ as the lattice constant of the artificial SL unit cell increases, due to the deposited heat.

### UXRD experiment at a plasma X-ray source

During the experiment at the table-top laser-driven plasma X-ray source (PXS) at University of Potsdam^58^ the sample was excited with p-polarized 100 fs laser pulses centered around 800 nm with an incident pump pulse energy of 167 μJ at 1 kHz repetition rate. The laser beam profile was approximately 0.9 × 0.8 mm^2^ (major and minor full-width half-maximum (FWHM) diameter) and therefore larger than the X-ray beam profile of approximately 0.3 × 0.3 mm^2^ (FWHM). The pump pulses were incident under 41.6^°^ with respect to the sample surface, which corresponds to an incident fluence of 13.5 mJ/cm^2^. For the sample structure and the excitation geometry, an optical transfer matrix model yields an absorption of 25%, resulting in an absorbed fluence of 3.3 mJ/cm^2^, while the remaining 75% are reflected. We tracked the time-resolved position of the (1 1 1) Bragg peaks of Cu and Ni at a fixed incidence angle^59^ of 21.6^°^ for the 200 fs X-ray probe pulses at a wavelength of 1.54 Å (Cu K_*α*_) generated by the PXS^58^. The diffracted X-rays were detected by an area detector (Dectris PILATUS 100 K). The layer-specific strain response is derived from the transient shift of the Bragg peak using a Gaussian fit.

### UXRD experiment at the MID instrument at EuXFEL

During the experiment at the Materials Imaging and Dynamics (MID) instrument at EuXFEL^45^ the sample was excited with p-polarized 50 fs laser pulses centered around 800 nm, having an incident pump pulse energy of 30 μJ or 60 μJ at a 10 Hz repetition rate. The laser beam profile was on the order of 0.35 × 0.4 mm^2^ (major and minor FWHM-diameter), much larger than the X-ray beam profile of approximately 15 × 15 μm^2^ (FWHM). The pump pulses were incident under 19^°^ with respect to the sample surface, which corresponds to an incident fluence of 6.5 mJ/cm^2^ and 13 mJ/cm^2^, respectively. For the different excitation angles compared to the PXS measurement, the optical transfer matrix model yields an absorption of 40%, resulting in an absorbed fluence of 2.6 mJ/cm^2^ and 5.2 mJ/cm^2^, respectively. We recorded the (1 1 1) Bragg peaks of Cu, Ni and the SL Bragg peaks SL (0 0 22) and SL (0 0 23) at a fixed incidence angle of 9^°^ as a function of the pump-probe delay using 50 fs X-ray probe pulses at a wavelength of 0.69 Å, i.e. 18 keV (SASE). The diffracted X-rays were detected by an area detector (AGIPD 1M^60^). The strain and intensity of each Bragg peak were derived from the transient shift and the area of the fitted Bragg peaks. To ensure a good intensity normalization, an area detector (ePix100) was used to measure the scattering from a Kapton target close to the sample. The intensity of the SL (0 0 22) Bragg peak exhibits significantly larger relative noise due to the smaller intensity changes. Error bars were omitted from the plot because the statistical error is considerably smaller than the measured intensity fluctuations prior to the excitation. These fluctuations likely stem from fluctuations in the X-ray spectrum and the limitation of the applied intensity normalization.

### Modeling UXRD experiment

We utilize the modular python library UDKM1DSIM^61^ that supports dynamical X-ray simulation, and include a temperature-dependent structure factor (Debye-Waller factor) to simulate the UXRD experiments. The thicknesses of the individual Pt and Cu layers of the SL in the simulation are adjusted to 11 atomic layers of Pt and 11 atomic layers of Cu to reproduce the measured diffraction pattern with the dynamical X-ray diffraction simulation (shown in Fig. 4). Using the complex refractive indices of each layer, the optical absorption profile is calculated for the appropriate angle of incidence and p-polarization. The optical transfer matrix method accounting for interference^62^ is implemented in the toolbox^61^. The absorbed energy density enters as the source term *S*_e_(*z*, *t*) in the differential equation of the d2TM^34^ with the rise-time of the 50 fs long laser pump pulses:

2 $${C}_{{{{\rm{e}}}}}({T}_{{{{\rm{e}}}}})\frac{\partial {T}_{{{{\rm{e}}}}}}{\partial t} 	=\frac{\partial }{\partial z}\left({\kappa }_{{{{\rm{e}}}}}({T}_{{{{\rm{e}}}}},{T}_{{{{\rm{ph}}}}})\frac{\partial {T}_{{{{\rm{e}}}}}}{\partial z}\right)+g({T}_{{{{\rm{ph}}}}}-{T}_{{{{\rm{e}}}}})+{S}_{{{{\rm{e}}}}}(z,t)\\ {C}_{{{{\rm{ph}}}}}\frac{\partial {T}_{{{{\rm{ph}}}}}}{\partial t} 	=\frac{\partial }{\partial z}\left({\kappa }_{{{{\rm{ph}}}}}\frac{\partial {T}_{{{{\rm{ph}}}}}}{\partial z}\right)+g({T}_{{{{\rm{e}}}}}-{T}_{{{{\rm{ph}}}}}).$$

The spatial coordinate *z* is perpendicular to the sample surface, pointing towards the substrate. The equations are coupled through the material-specific ab-initio electron-phonon coupling constant *g*(*T*_e_), which transfers energy from the electron system described by the ab initio electronic specific heat *C*_e_(*T*_e_) (see Fig. 3b) to the phonon system with a constant specific heat *C*_ph_. The electronic and phononic thermal conductivities *κ*_e/ph_ and all other literature parameters are listed in Supplementary Table 1. For a correct description of the non-equilibrium transport, i.e. *T*_e_ ≫ *T*_ph_ we have to consider the dependence of the electronic heat conductivity ${\kappa }_{{{{\rm{e}}}}}={\kappa }_{{{{\rm{e}}}}}^{0}\,{T}_{{{{\rm{e}}}}}/{T}_{{{{\rm{ph}}}}}$ on the temperatures of the electron and phonon systems^25,28,63–65^. Here, ${\kappa }_{{{{\rm{e}}}}}^{0}$ represents the empirical equilibrium conductivity^25,66^. In the first picoseconds, the electronic stress ${\sigma }_{{{{\rm{e}}}}}={\Gamma }_{{{{\rm{e}}}}}\Delta {\rho }_{{{{\rm{e}}}}}^{Q}$ is highly relevant. Their energy density is given by $\Delta {\rho }_{{{{\rm{e}}}}}^{Q}={C}_{{{{\rm{e}}}}}({T}_{{{{\rm{e}}}}})\Delta {T}_{{{{\rm{e}}}}}$. Although a diffusive model for electrons and phonons in nanometric thin films below the diffusion length is a crude approximation^21,22^, we capture the essential physics correctly. More detailed modeling of electron transport would, in any case, result in a nearly uniform electron temperature across the SL, on timescales much shorter than the experimental time resolution. This nearly constant electron temperature immediately implies a sharply structured electronic energy density that drives the observed THz phonon. The spatiotemporal temperature maps from the d2TM are used to calculate the spatiotemporal strain dynamics. The material- and subsystem-specific Grüneisen parameters Γ_e/ph_ linearly relate the calculated spatiotemporal energy densities to the stress contribution of the respective subsystem (see Supplementary Fig. 2). Their superposition serves as an input for a linear masses-and-springs model^57^ considering the laterally homogeneous excitation and Poisson stress of thin films on ultrafast timescales^34^. The linear masses-and-springs model is even appropriate for ultrathin heterostructures, but it is fully consistent with the elastic wave equation from continuum elasticity theory:

3 $${\rho }_{{{{\rm{m}}}}}\frac{{\partial }^{2}u}{\partial {t}^{2}}=\frac{\partial }{\partial z}{\sigma }_{z}^{{{{\rm{tot}}}}}=\frac{\partial }{\partial z}({c}_{3333}\cdot \eta -{\sigma }_{{{{\rm{e}}}}}-{\sigma }_{{{{\rm{ph}}}}}),$$

with the mass density *ρ*_m_, the atomic displacement *u* and the total stress ${\sigma }_{z}^{{{{\rm{tot}}}}}$ that comprises the elastic constants *c*, the strain *η* and the electron and phonon stresses *σ*_e/ph_. From the resulting strain map (see Supplementary Fig. 2) and the spatiotemporal phonon temperature, the diffraction curve was calculated by dynamical X-ray diffraction theory. The spatiotemporal phonon temperature is used to calculate the Debye-Waller factor, which reduces the X-ray structure factor via the Debye parameter *B*(*T*) = 8/3 *π*^2^〈*u*^2^〉 that is tabulated for different temperatures^67^. The simulated diffraction pattern is folded with a broad Gaussian to mimic the large mosaicity of the sample. The result is fitted in the same way as the experimental data to extract the strain and intensity for each Bragg peak shown in Figs. 1, 2. To describe the different excitation strengths in the experiment, we apply the same set of parameters and only adjust the source term in the d2TM according to the experiment.

### First-principles calculations of mode-specific electron-phonon coupling

The large group velocity of the s-p conduction electrons in Cu is mismatched with that of the heat-carrying electrons (d-band electrons) in Pt, which leads to a large reflection coefficient of the electron wave at the interface. Thus, the electron stress can be interpreted as a momentum transfer to the lattice. In order to confirm that it is electron pressure that drives the observed modes, we perform ab-initio modeling of the electron-phonon coupling in the two relevant materials.

In contrast to semi-empirical *g*(*T*_e_) parameterizations^47^, we compute the mode-resolved electron-phonon coupling *G*_*qν*_ for fcc Pt and Cu (see Fig. 3a) from first principles, using the Eliashberg-theory formulation^68,69^ and derive a lower bound for the effective energy relaxation time *τ*_eff_. We used the Quantum Espresso package^70^ and EPW code^71^ with the generalized gradient approximation^72^ for the exchange-correlation functional and fully relativistic, norm-conserving pseudopotentials^73^ accounting for spin-orbit coupling in Pt. The Brillouin zone was sampled with a 16 × 16 × 16 *k*-point mesh, and dynamical matrices were computed on a 4 × 4 × 4 *q*-point grid, using a plane-wave kinetic energy cut-off of 120 Ry. Maximally localized Wannier functions were constructed from the Pt d and Cu s–d orbitals. Electron-phonon coupling for Pt and Cu was evaluated on dense 40 × 40 × 40 *k*-point and 20 × 20 × 20 *q*-point meshes via Wannier interpolation, as implemented in EPW^71^. Lattice parameters were set to the experimental room-temperature values for Pt and Cu, and internal coordinates were fully relaxed. Figure 3a shows the calculated mode-resolved electron-phonon coupling *G*_*qν*_ on the phonon dispersions for fcc Pt. The color-coded coupling constant *G*_*qν*_ is maximal for transverse or longitudinal acoustic phonons near the Brillouin-zone boundary, ranging from 12 to 22 meV corresponding to phonon frequency of *ν* ≈ 3 to 5.5 THz. In contrast, long-wavelength modes near *Γ* (including the longitudinal acoustic  ~ 1 THz mode relevant to the coherent SL vibration) exhibit couplings that are significantly smaller. This indicates that the electron-phonon interaction is too weak to drive the observed coherent oscillations. For comparison to the d2TM, we calculated the mode-averaged electron-phonon coupling constant *g*(*T*_e_) = 1/*N*_*q*_ ∑_*qν*_ *G*_*qν*_(*T*_e_), where *T*_e_ enters through material-specific, temperature-dependent electron occupations and *T*_ph_ = 300 K. The calculated *g*(*T*_e_) for Pt and Cu are shown in Fig. 3b as a function of *T*_e_. Although the ab initio temperature dependences of *g*_Pt_ and *g*_Cu_ compare favorably to the semi-empirical function^47^, its magnitude is about two times smaller for Pt and about twice larger for Cu. The relaxation of the combined Pt/Cu electron system to phonons is dominated by the fast effective electron-phonon relaxation time *τ*_eff_ = *C*_e,Pt_(*T*_*e*_)/*g*_Pt_(*T*_e_) > 1 ps in the relevant temperature range. Crucially, even this lower bound of the relaxation time is already too long for phonon-induced stress to play a significant role in actuating the 1 THz phonon. Here, *C*_e_(*T*_e_) is the computed electronic specific heat (Fig. 3b). The resulting material-averaged *τ*_eff_ gives the overall electron-phonon energy-transfer rate relevant to d2TM fits, but it does not correspond to exciting a selected single phonon mode. Because *g* is dominated by high-frequency LA modes near  ~ 4 THz (16 meV) in Pt (Fig. 3a), *τ*_eff_ constitutes a lower bound for the mode-specific transfer time of the coherent 1 THz superlattice phonon, *τ*_1 THz_ ≫ *τ*_eff_. This distinction emphasizes why the 1 THz mode cannot be efficiently driven by conventional incoherent electron-phonon coupling, because already *τ*_eff_ > 1 ps.

## Supplementary information

Supplementary Information

Transparent Peer Review file

## Data Availability

Data recorded for the experiment at the European XFEL are available at 10.22003/XFEL.EU-DATA-003491-00. Supporting data are available at 10.5281/zenodo.19463085 and from the corresponding authors upon request.

## Code availability

The codes used to evaluate the data and to start the d2TM modeling are saved together with the data at 10.22003/XFEL.EU-DATA-003491-00 and at 10.5281/zenodo.19463085. The code of the udkm1dsim toolbox used for modeling is available at https://github.com/dschick/udkm1Dsim, version 2.3.0.
