# Supplementary material for: Electron pressure drives THz phonons in metal–metal superlattices
Source: Nat Commun. 2026 Jun 16;17:5308. doi: 10.1038/s41467-026-73927-y (PMC13273064; doi:10.1038/s41467-026-73927-y)
Supplement: Supplementary file 1 — Supplementary Information [file 41467_2026_73927_MOESM1_ESM.pdf]

Supplementary information to "Electron pressure drives THz phonons in metal–metal superlattices"

Jan-Etienne Pudell,[1] Maximilian Mattern,[2,3] Marc Herzog,[2] Alexander von Reppert,[2] Chandan K. Singh,[4] Daniel Schick,[3] Michel Hehn,[5] Ulrike Boesenberg,[1] Angel Rodriguez-Fernandez,[1] Roman Shayduk,[1] Wonhyuk Jo,[1] Johannes Möller,[1] Jörg Hallmann,[1] James Wrigley,[1] Peter M. Oppeneer,[4] Anders Madsen,[1] and Matias Bargheer[2,6]

[1]*European X-ray Free-Electron Laser Facility, 22869 Schenefeld, Germany*
[2]*Institut für Physik und Astronomie, Universität Potsdam, 14476 Potsdam, Germany*
[3]*Max-Born-Institut (MBI) im Forschungsverbund Berlin e.V., 12489 Berlin, Germany*
[4]*Department of Physics and Astronomy, Uppsala University, P.O. Box 516, SE-75120 Uppsala, Sweden*
[5]*Institut Jean Lamour (UMR CNRS 7198), Université Lorraine, 54000 Nancy, France*
[6]*Helmholtz-Zentrum Berlin für Materialien und Energie GmbH, Wilhelm-Conrad-Röntgen Campus, BESSY II, 12489 Berlin, Germany*

(Dated: May 14, 2026)

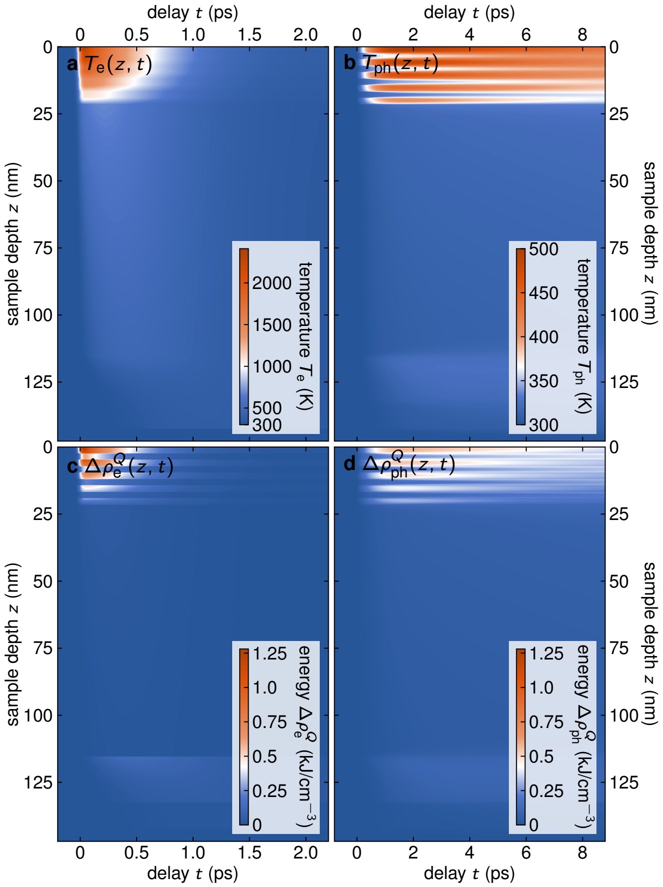

Supplementary Figure 1 | Results of the diffusive two-temperature model (d2TM): a, Electron temperature T_e and **b**, phonon temperature T_ph within the heterostructure for an excitation of 2.6 mJ/cm^2 (absorbed) with a pulse duration of 50 fs. **d**, Corresponding energy density change of electrons $\Delta\rho_\mathrm{e}^Q$ and **e** energy density change of phonons $\Delta\rho_\mathrm{ph}^Q$.

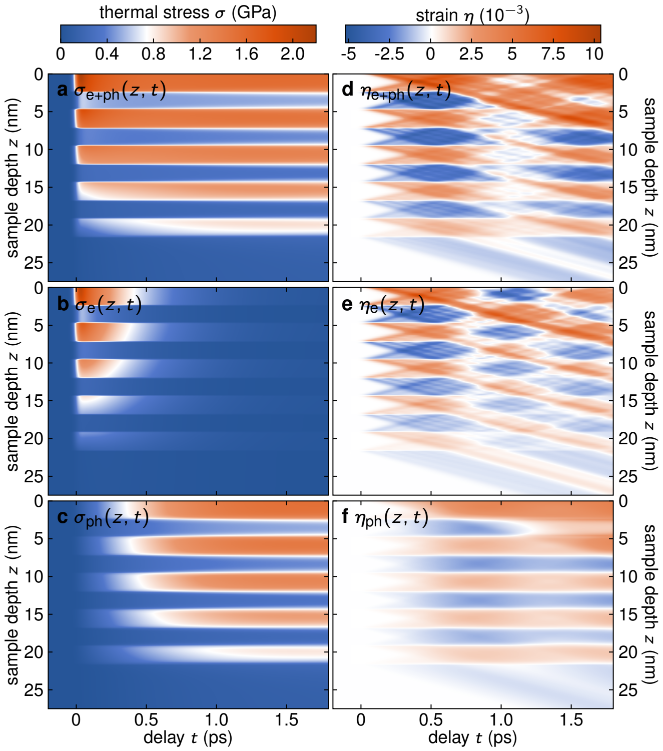

Supplementary Figure 2 | Spatio-temporal evolution of thermal stress and resulting strain: a, total simulated stress of electron-phonon system; **b,c,** electron and phonon contributions, respectively. **d-f,** corresponding strain response. The THz phonon mode is driven by the electronic stress (**b,e**), whereas the phonon contribution induces a non-oscillating expansion of the Pt layers accompanied by a compression of Cu, which relaxes via an acoustic wave.

Supplementary Table 1 | Thermophysical parameters of Pt, Cu, Ni, Ta and the glass substrate

	Pt	Cu SL	Cu	Ni	Ta	glass
γ^{S} (μJ cm^{-3}K^{-2})	740[1]	98	98[1]	1065[1]	380[2]	–
C_{ph} (J cm^{-3}K^{-1})	2.85[3]	3.44	3.44	3.94[4]	2.33[2]	1.80[5]
κ_e^0 (W m^{-1}K^{-1})	66[6] (23)	140	396[1] (475)	81[1,7]	52	–
κ_{ph} (W m^{-1}K^{-1})	5[6] (0.25)	0.25	5	10[7]	5	1[5]
g (PW m^{-3}K^{-1})	1000† (450†)	70†	70†	360[8]	100	–
ρ (g cm^{-3})	21.45	8.96	8.96	8.91	16.68	2.54[5]
v_{s} (nm ps^{-1})	4.2[9,10]	5.2	5.2[11,12]	6.3[13]	4.2[14]	5.7[5]
n	$2.7 + 6.1$i[15]	$0.11 + 5.1$i	$0.11 + 5.1$i[16]			
Γ_{e}	2.4[17](1.6)	0.9	0.9[17]	1.4[18]	1.3[17]	–
Γ_{ph}	2.6[19]	2.0	2.0[20]	1.8[18]	1.6[17]	0.3[5]

Thermophysical parameters of Pt, Cu, Ni, Ta and the glass substrate taken from the indicated literature. Values in brackets are optimised values for the simulation. The thermal conductivities of Pt and Cu inside the SL are reduced considerably, this is reasonable due to the interfaces between each Cu and Pt layer[21]. For the simulation, temperature-dependent electron-phonon coupling constants and electronic heat capacities for Cu and Pt were used. The average value is given in the table and marked with †. The parametrized Debye parameter B was taken from Peng *et al.* [22] for temperatures larger than 80 K.

[1] J. Hohlfeld, S.-S. Wellershoff, J. Güdde, U. Conrad, V. Jähnke, and E. Matthias, Electron and lattice dynamics following optical excitation of metals, Chemical Physics **251**, 237 (2000).

[2] V. Y. Bodryakov and A. N. Bashkatov, Heat capacity of tantalum in the normal and superconducting states: Identification of the contributions, Russian Metallurgy (Metally) **2013**, 671 (2013).

[3] R. Shayduk, V. Vonk, B. Arndt, D. Franz, J. Strempfer, S. Francoual, T. F. Keller, T. Spitzbart, and A. Stierle, Nanosecond laser pulse heating of a platinum surface studied by pump-probe X-ray diffraction, Applied Physics Letters **109**, 043107 (2016).

[4] P. J. Meschter, J. W. Wright, C. R. Brooks, and T. G. Kollie, Physical contributions to the heat capacity of nickel, Journal of Physics and Chemistry of Solids **42**, 861 (1981).

[5] C. Incorporated, Corning 1737 AMLCD Glass, (2002).

[6] M. J. Duggin, The thermal conductivities of aluminium and platinum, Journal of Physics D: Applied Physics **3**, 21 (1970).

[7] X. Zheng, D. Cahill, P. Krasnochtchekov, R. Averback, and J. Zhao, High-throughput thermal conductivity measurements of nickel solid solutions and the applicability of the Wiedemann–Franz law, Acta Materialia **55**, 5177 (2007).

[8] Z. Lin, L. V. Zhigilei, and V. Celli, Electron-phonon coupling and electron heat capacity of metals under conditions of strong electron-phonon nonequilibrium, Physical Review B **77**, 776 (2008).

[9] R. E. Macfarlane, J. A. Rayne, and C. K. Jones, Anomalous temperature dependence of shear modulus c44 for platinum, Physics Letters **18**, 91 (1965).

[10] S. M. Collard and R. B. McLellan, High-temperature elastic constants of platinum single crystals, Acta Metallurgica et Materialia **40**, 699 (1992).

[11] F. A. A. Radwan, Some Properties of Copper-Gold and Silver Gold Alloys at Different % of Gold, World Congress on Engineering 2012. July 4-6, 2012. London, UK. , 1221 (2010).

[12] H. M. Ledbetter and E. R. Naimon, Elastic Properties of Metals and Alloys. II. Copper, Journal of Physical and Chemical Reference Data **3**, 897 (1974).

[13] J. R. Neighbours, F. W. Bratten, and C. S. Smith, The Elastic Constants of Nickel, Journal of Applied Physics **23**, 389 (1952).

[14] F. H. Featherston and J. R. Neighbours, Elastic Constants of Tantalum, Tungsten, and Molybdenum, Physical Review **130**, 1324 (1963).

[15] A. Tselin, M. Pogodaeva, S. Levchenko, A. Kalmykov, K. Garbuzov, A. Smirnov, and V. Drachev, Exploring the dielectric function of platinum, Physical Review B **110**, 195130 (2024).

[16] K. M. McPeak, S. V. Jayanti, S. J. P. Kress, S. Meyer, S. Iotti, A. Rossinelli, and D. J. Norris, Plasmonic Films Can Easily Be Better: Rules and Recipes, ACS Photonics **2**, 326 (2015).

[17] R. S. Krishnan, R. Srinivasan, S. Devanarayanan, and B. R. Pamplin, *Thermal Expansion of Crystals: International Series in The Science of The Solid State* (Elsevier Science, Burlington, 1979).

[18] X. Wang, S. Nie, J. Li, R. Clinite, M. Wartenbe, M. Martin, W. Liang, and J. Cao, Electronic Grüneisen parameter and thermal expansion in ferromagnetic transition metal, Applied Physics Letters **92**, 121918 (2008).

[19] F. C. Nix and D. MacNair, The Thermal Expansion of Pure Metals. II: Molybdenum, Palladium, Silver, Tantalum, Tungsten, Platinum, and Lead, Physical Review **61**, 74 (1942).

[20] F. C. Nix and D. MacNair, The Thermal Expansion of Pure Metals: Copper, Gold, Aluminum, Nickel, and Iron, Physical Review **60**, 597 (1941).

[21] M. Herzog, A. von Reppert, J.-E. Pudell, C. Henkel, M. Kronseder, C. H. Back, A. A. Maznev, and M. Bargheer, Phonon-Dominated Energy Transport in Purely Metallic Heterostructures, Advanced Functional Materials **32**, 2206179 (2022).

[22] L.-M. Peng, G. Ren, S. L. Dudarev, and M. J. Whelan, Debye–Waller Factors and Absorptive Scattering Factors of Elemental Crystals, Acta Crystallographica Section A: Foundations of Crystallography **52**, 456 (1996).
