# Supplementary material for: Electron pressure drives THz phonons in metal–metal superlattices
Source: Nat Commun. 2026 Jun 16;17:5308. doi: 10.1038/s41467-026-73927-y (PMC13273064; doi:10.1038/s41467-026-73927-y)
Supplement: Supplementary file 2 — Transparent Peer Review file [file 41467_2026_73927_MOESM2_ESM.pdf]

nature portfolio

Peer Review File

Electron pressure drives THz phonons in metal–metal superlattices

Corresponding Author: Professor Matias Bargheer

This manuscript has been previously reviewed at another journal. This document only contains information relating to versions considered at Nature Communications.

Attachments originally included by the reviewers as part of their assessment can be found at the end of this file.

Version 0:

Reviewer comments:

Reviewer #1

(Remarks to the Author)
I am in favor of publication of this work in Nature Communications - the results are interesting and impactful and will be of interest to the broad readership of this journal. However I think there are still a number of issues, misinterpretations, and incorrect statements in the current version which absolutely need to be fixed.

1. The authors seem to be implying that the force which drives the measured lattice expansion comes from a direct interaction of the electrons with the interfaces. This is not correct - there is a force applied throughout the excited material. The same is true in semiconductors, the much more well studied example. See for example Thomsen et al, PRB 34, 4129 (1986) where in the simplest case of a temperature-jump induced force / stress, this force acts over the region defined by the optical penetration depth. Similarly in the case of a metal-metal superlattice, the force (no matter what type of electron-phonon coupling is important) is not just a force on the interfaces. The authors imply the opposite. For example they state:

"Whether such electronic stress can form across purely metallic interfaces remains unclear, because experimental evidence is missing. In metals, electrons are generally considered free, yet potential steps and band-structure mismatch at interfaces could localise electronic states. If this localisation were strong enough, it could generate an electron-pressure gradient capable of driving coherent lattice oscillations in a fully metallic metamaterial."

2. This phrasing above is additional confusing because for a thermally generated stress (and even one that turns on on a time-scale longer than the superlattice period), one will still drive coherent lattice oscillations. It's true that if the coupling time is longer than the effective phonon period this coupling will be damped but it is not zero. One can see this in the simplest case of a forced simple harmonic oscillator where if a time dependent force turns on on a time-scale longer than the period of the oscillator, the oscillator still responds and oscillates at its fundamental frequency. In their response letter and in the paper the authors imply on the contrary that because theory indicates longer coupling times for the 'ordinary' e-p coupling, this cannot play a role. For example:

"To actuate a coherent and narrowband strain wave with T = 1 ps period, the spatially periodic stress modulation inside the SL must develop much faster than T, which is faster than the effective time scale $\tau_{eff} \approx 1$ ps of incoherent energy relaxation between electrons and phonons in these metals." Again a forced simple harmonic oscillator model with a slowly developing stress turn on time is all one needs to see that this is incorrect.

To make a statement regarding which type of e-p coupling is driving the coherent response then becomes a quantitative argument about the magnitude of the force and the magnitude of the observed displacement. As one suggestion, if the authors were able to extract an effective Gruneisen parameter (as they discuss on page 4) which made quantitative sense this would support their argument.

3. The authors state in their response "Although there is 30 years of publication on deformation potential interaction in semiconductors and semiconductor superlattices (or metal/semiconductor), all my interactions with members of the phonons community indicated that it is surprising to drive

metal-metal superlattices with giant amplitude by electron-pressure." I do not find this a convincing argument - if this is true the authors should support this argument in the paper in a quantitative way. Similarly in their response to ref2 it is stated "It is not obvious to researchers in experimental science and technology that electrons should exert a pressure at metal/metal interfaces." This needs to be supported not by polling researchers but based on a quantitative argument.

In summary, I think the results are interesting and support publication. Indeed, I actually think it is likely the case that the authors are correct and that the electronic pressure is dominant. However this needs to be justified and in the current version this has not been achieved.

Version 1:

Reviewer comments:

Reviewer #1

(Remarks to the Author)
The authors have carefully addressed my comments I am in favor of publication of this work in Nature Communications.

Open Access This Peer Review File is licensed under a Creative Commons Attribution 4.0 International License, which permits use, sharing, adaptation, distribution and reproduction in any medium or format, as long as you give appropriate credit to the original author(s) and the source, provide a link to the Creative Commons license, and indicate if changes were made.
In cases where reviewers are anonymous, credit should be given to 'Anonymous Referee' and the source.
The images or other third party material in this Peer Review File are included in the article's Creative Commons license, unless indicated otherwise in a credit line to the material. If material is not included in the article's Creative Commons license and your intended use is not permitted by statutory regulation or exceeds the permitted use, you will need to obtain permission directly from the copyright holder.
To view a copy of this license, visit https://creativecommons.org/licenses/by/4.0/

We have provided a new manuscript with red highlighting of all changes made to the manuscript.

Point by point answer to the reviewer's comments:

REVIEWER COMMENTS

Reviewer #1 (Remarks to the Author):

I am in favor of publication of this work in Nature Communications - the results are interesting and impactful and will be of interest to the broad readership of this journal.

➔ Thank you very for taking the time to study our manuscript and for this positive assessment!

However I think there are still a number of issues, misinterpretations, and incorrect statements in the current version which absolutely need to be fixed.

1. The authors seem to be implying that the force which drives the measured lattice expansion comes from a direct interaction of the electrons with the interfaces. This is not correct - there is a force applied throughout the excited material. The same is true in semiconductors, the much more well studied example. See for example Thomsen et al, PRB 34, 4129 (1986) where in the simplest case of a temperature-jump induced force / stress, this force acts over the region defined by the optical penetration depth. Similarly in the case of a metal-metal superlattice, the force (no matter what type of electron-phonon coupling is important) is not just a force on the interfaces. The authors imply the opposite. For example they state:

"Whether such electronic stress can form across purely metallic interfaces remains unclear, because experimental evidence is missing. In metals, electrons are generally considered free, yet potential steps and band-structure mismatch at interfaces could localise electronic states. If this localisation were strong enough, it could generate an electron-pressure gradient capable of driving coherent lattice oscillations in a fully metallic metamaterial."

➔ Thank you for spotting this nuance! Indeed, the cited paragraph is only correct, if we write:
Whether such electronic stress **gradients** can form across purely metallic interfaces remains unclear, because experimental evidence is missing. In metals, electrons are generally considered free, yet potential steps and band-structure mismatch at interfaces could localise electronic states.

➔ Since it is important to show why metallic and semiconducting/insulating materials need to be distinguished, we rephrased the paragraph before "Here we demonstrate", essentially by reordering arguments. We thank the reviewer for making us improve the wording:

"Electron pressure can be the dominant stress on sub-picosecond timescales. Already in semiconductors, photoexcited carriers can propagate and thereby generate stress profiles that deviate from the optical absorption profile [34,36]. In metals, however, rapid electronic transport delocalizes the deposited energy and suppresses steep electronic-stress gradients. For nonmetallic multilayer systems [37] and at metal surfaces [38–42], the pressure gradients at the interfaces efficiently drive structural

motion. Whether electronic stress gradients can form across purely metallic interfaces remains unclear, because experimental evidence is missing. In metal heterostructures, electrons are generally considered free, yet potential steps and band-structure mismatch between different metals could localise electronic states. If this localisation were strong enough, it could generate an electron-pressure gradient at the interface between two metals capable of driving coherent lattice oscillations in a fully metallic superlattices."

2. This phrasing above is additional confusing because for a thermally generated stress (and even one that turns on on a time-scale longer than the superlattice period), one will still drive coherent lattice oscillations. It's true that if the coupling time is longer than the effective phonon period this coupling will be damped but it is not zero. One can see this in the simplest case of a forced simple harmonic oscillator where if a time dependent force turns on on a time-scale longer than the period of the oscillator, the oscillator still responds and oscillates at its fundamental frequency. In their response letter and in the paper the authors imply on the contrary that because theory indicates longer coupling times for the 'ordinary' e-p coupling, this cannot play a role. For example:

"To actuate a coherent and narrowband strain wave with T = 1 ps period, the spatially periodic stress modulation inside the SL must develop much faster than T, which is faster than the effective time scale $\tau_{eff} \approx 1$ ps of incoherent energy relaxation between electrons and phonons in these metals." Again, a forced simple harmonic oscillator model with a slowly developing stress turn on time is all one needs to see that this is incorrect.

To make a statement regarding which type of e-p coupling is driving the coherent response then becomes a quantitative argument about the magnitude of the force and the magnitude of the observed displacement. As one suggestion, if the authors were able to extract an effective Gruneisen parameter (as they discuss on page 4) which made quantitative sense this would support their argument.

- ➔ Thank you for explaining that this was unclear. Indeed our model takes into account both electron and phonon stress (eq. 2) and hence also stress gradients. Moreover, the electron-phonon coupling constant $g(T_e)$ that was calculated ab initio for both materials enters the two temperature model (eq. 1) which describes precisely the electron-phonon coupling that leads to the "thermally generated stress" the reviewer mentions. Extended data Table 1 contains the Grüneisen constants that we use in the model. We do not extract the Grüneisen constant, as we do not want to overemphasize the slight adjustments that are indicated in the table just to be accurate.
- ➔ We hope to convince the reviewer by bringing to the attention Fig. 2:

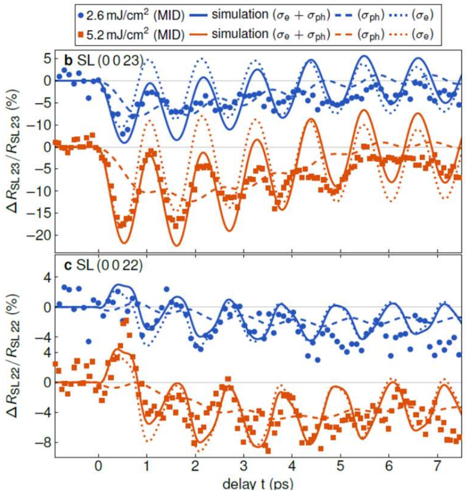

tions. **b**, Transient intensity change of the (0 0 23) SL Bragg peak measured at the MID instrument for two different absorbed fluences (2.6 mJ/cm^2 [blue] and 5.2 mJ/cm^2 [orange]). **c**, same for (0 0 22). The lines originate from a simulation of the elastic response to the stresses σ_e from electrons and/or σ_{ph} from phonons. The simulation assumes a perfect sample. Including a phenomenological phonon damping or dephasing would yield an even better match to the measured data. The strain response to individual contributions of only electron or phonon stress are shown as dotted or dashed lines, respectively, confirming that the 1 THz oscillation must be driven by electron stress.

- ➔ We adjust the sentence as: “To actuate a coherent and narrowband strain wave with T = 1 ps period efficiently and with large amplitude, the spatially periodic stress modulation inside the SL must develop much faster than T…”
- ➔ We now sharpened the Figure caption to emphasize that there are three lines compared to the measured Data:
- ➔ **b,** Transient intensity change of the (0 0 23) SL Bragg peak measured at the MID instrument for two different absorbed fluences (2.6 mJ/cm2 [blue] and 5.2 mJ/cm2 [orange]). **c,** same for (0 0 22). The lines originate from simulations of the elastic response to the stresses σ_e from electrons (dotted) or σ_ph from phonons (dashed) and finally to the sum of both stresses (solid lines). Taking into account both contributions yields the best agreement. The large amplitude of the 1 THz oscillation is only reproduced if a significant electron stress is included, whereas phonon stress mainly dominates the slow, non-oscillatory response. The simulation assumes a perfect sample. Including a phenomenological phonon damping or dephasing would yield an even better match to the measured data.

3. The authors state in their response "Although there is 30 years of publication on deformation potential interaction in semiconductors and semiconductor superlattices (or metal/semiconductor), all my interactions with members of the phonons community indicated that it is surprising to drive
metal-metal superlattices with giant amplitude by electron-pressure." I do not find this a convincing argument - if this is true the authors should support this argument in the paper in a quantitative way. Similarly in their response to ref2 it is stated "It is not obvious to researchers in experimental science and technology that electrons should exert a pressure at metal/metal interfaces." This needs to be supported not by polling researchers but based on a quantitative argument.

- ➔ We agree that without a poll one cannot make a scientifically sound statement about how surprising this is. I think that I just wanted to set my experience from discussions with colleagues against the statements of the two reviewers.

In summary, I think the results are interesting and support publication. Indeed, I actually think it is likely the case that the authors are correct and that the electronic pressure is dominant. , this needs to be justified and in the current version this has not been achieved.

- ➔ We hope that by our above comments we can convince the reviewer that our modeling is solid. In particular our additional emphasis of the dashed and dotted lines in Fig. 2b and c, which clearly indicate that the electronic pressure is dominant, should resolve the uncertainties.
- ➔ We summarize the steps of the modeling here once more:
  a) We calculate the electronic specific heat and the e-ph interaction in Pt and Cu. This is done in a mode-specific way to show that at 1 THz the intrinsic coupling is particularly weak.
  b) We use these ab-initio values in our diffusive 2TM (eq. 2) to model the spatio-temporal temperature profile (energy density profile) in the electron and phonon system separately.
  c) From the energy densities we calculate the spatio-temporal electron and phonon stress profiles within the Grüneisen model.
  d) These stresses enter an elastic wave equation (eq. 2) that describes the atomic motion, fully consistent with Thomsen+Grahn 1986. The connection was detailed in M. Mattern, et al., Photoacoustics 31, 100503 (2023).
  e) From the calculated atomic motion, which shows a large-amplitude coherent longitudinal 1 THz phonon, we calculate the UXRD pattern.
  f) The best agreement is achieved for combined electron AND phonon stress, however, the by far dominant contribution to the 1 THz mode amplitude originates from electron stress (see Fig. 2 reproduced above)
  g) As an additional illustration we added the spatio-temporal strain profiles in the extended data and moved a figure on sample characterization from the extended data to the methods section, where it is useful to illustrate the connection of the measured x-ray intensity to the strain amplitude.
